# A Real-Time Monitoring System of Industry Carbon Monoxide Based on Wireless Sensor Networks

**DOI:** 10.3390/s151129535

**Published:** 2015-11-20

**Authors:** Jiachen Yang, Jianxiong Zhou, Zhihan Lv, Wei Wei, Houbing Song

**Affiliations:** 1 School of Electronic Information Engineering, Tianjin University, 92 Weijin Road, Tianjin 300072, China; E-Mails: yangjiachen@tju.edu.cn (J.Y.); zhoujx@tju.edu.cn (J.Z.); 2 Shenzhen Institute of Advanced Technology, Chinese Academy of Sciences, 1068 Xueyuan Avenue, Shenzhen University Town, Shenzhen 518055, China; E-Mail: zh.lv@siat.ac.cn; 3 School of Computer Science and Engineering, Xi’an University of Technology, Xi’an 710048, China; E-Mail: weiwei@xaut.edu.cn; 4 Department of Electrical and Computer Engineering, West Virginia University, Montgomery, WV 25136, USA

**Keywords:** CO detection, real-time monitor, sensor networks, Wifi

## Abstract

Carbon monoxide (CO) burns or explodes at over-standard concentration. Hence, in this paper, a Wifi-based, real-time monitoring of a CO system is proposed for application in the construction industry, in which a sensor measuring node is designed by low-frequency modulation method to acquire CO concentration reliably, and a digital filtering method is adopted for noise filtering. According to the triangulation, the Wifi network is constructed to transmit information and determine the position of nodes. The measured data are displayed on a computer or smart phone by a graphical interface. The experiment shows that the monitoring system obtains excellent accuracy and stability in long-term continuous monitoring.

## 1. Introduction

CO is a colorless, odorless gas with molecular mass equal to the atmosphere, which is present indoors and outdoors [1]. CO will explode and burn in the condition of improper storage. Moreover, CO is an excellent fuel. Compared with coal, CO does not produce dust and sulfur during combustion and it is easy for transportation. For the coal-producing countries, it is a wise choice to choose CO as fuel. However, CO will explode at the concentration from 12.5% to 74.5% and burn beyond the concentration of 75% in the air in case of fire. Also, CO can directly affect the health of people. After being inhaled into the respiratory system, it will eventually prohibit hemoglobin (Hb) in blood cells binding and carrying oxygen molecules because it reacts with hemoglobin faster than oxygen does [2,3].

In order to reduce the harm of CO, it is meaningful to monitor the concentration of CO in a timely manner. So, CO monitoring can be considered as a promising practical application, which combines a Wireless Sensor Network (WSN) with the Internet of Things [4,5]. A wireless sensor network provides real-time monitoring of multiple distributed nodes, and then predicts environmental conditions in the area, which creates new opportunities for chemical and biological sensor systems in healthcare, environmental monitoring, process and quality control, and chemical and biological threat detection.

Wifi-based WSN is an emerging aspect of wireless technology, which consists of several nodes and access points. Wifi is suitable for industrial monitoring with the advantages of fast transmission rate and long transmission distance. In summary, Wifi-based WSNs are opening new markets for distributed sensors and sensor networks [6].

There are many detectable methods for sensor nodes. Currently, the United States Environmental Protection Agency (U.S. EPA) recommends non-dispersive infrared (NDIR) as a standard reference method. The principle of this technology is based on absorption of infrared radiation by CO molecules in the wavelength of 4.6 μm region [7]. This method has the advantages of not being easily affected by temperature, fast response and high sensitivity [8]. We have made some progress in the carbon dioxide concentration detected based on NDIR technology, and the accuracy of carbon dioxide concentration monitoring device by using pyroelectric sensor can be up to 0.23 mmHg.

In this study, a new hardware platform for CO sensor nodes is developed based on NDIR technology. To prevent jittering, a low frequency modulation method is proposed. Wireless communication between each sensor node and routers is fulfilled through a Wifi wireless network which can locate every sensor node and transport data from sensor node to PC or smartphone through routers. The sensor nodes and wireless network constitute a wireless sensor network,which is used in industrial plants in the storage of CO, and we can determine the location and concentration of CO leak points by the transmitted data nodes. The targeted experiments have demonstrated that this system has a strong stability.

The remainder of this paper is organized as follows. Section 2 presents a hardware solution to the system. The corresponding networks architecture for system is described in Section 3. We show experimental results and provide analysis in Section 4. And the study is concluded in Section 5.

## 2. System Hardware

**Figure 1:**
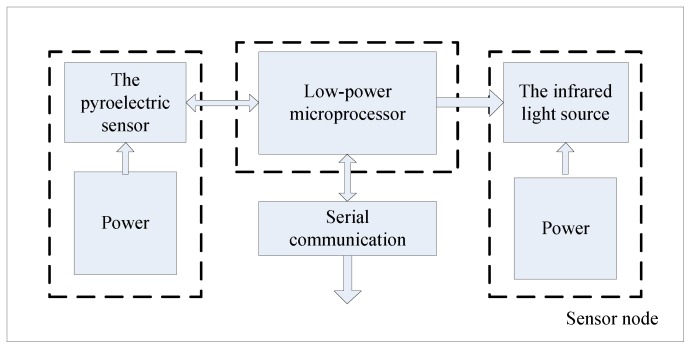
Sensor node monitoring modules.

**Figure 2:**
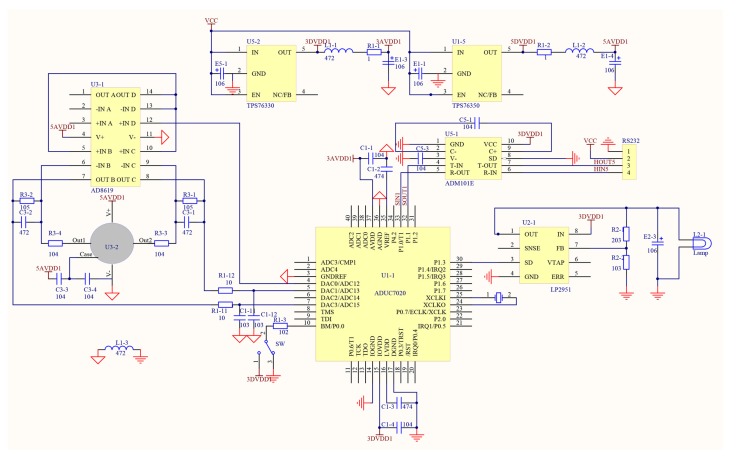
Circuit schematics of sensor node.

**Figure 3:**
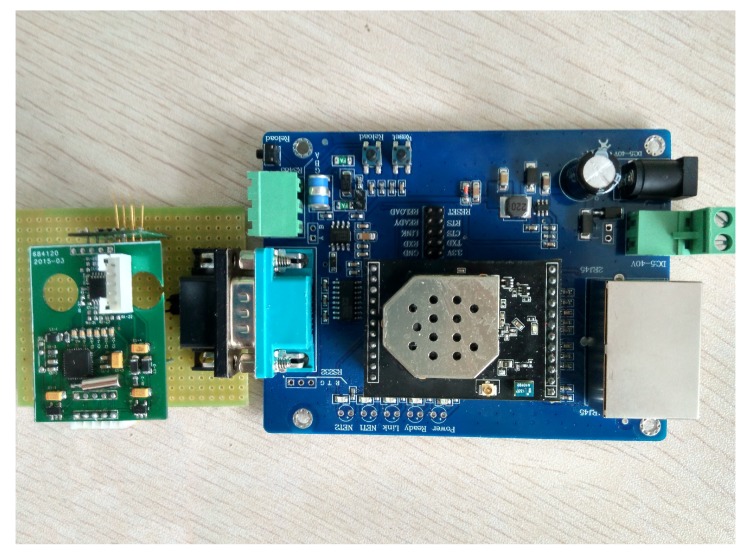
Actual picture of system hardware.

As the monitoring systems are often applied in the industrial and flammable gas detection, the detection module should be small and easy to carry and transport. Moreover, considering the explosion hazard of CO, we need a fast response system with precise detection node. In view of these reasons, the monitoring module adopts pyroelectric sensor, and the sensor node mainly comprises airway tube, CO concentration measurement module. Figure 1 shows the main monitoring modules, and the circuit schematics of sensor node are shown in Figure 2. In Figure 3, the actual picture of system hardware is displayed.

The new material possesses the pyroelectric sensor of a high precision and its thermal response time which is about 150 ms makes the sensor have a fast response rate, real-time monitoring. Hence,this sensor can satisfy the needs of CO monitoring. CO concentration is measured in accordance with Lambert-Beer Law [9], also, dual pyroelectric sensor can reduce external interference:

(1) $$I=I_{0}e^{-\alpha (\lambda )CL}$$

where *I* is the intensity of light striking the detector (*I*, ${W/\mathrm{cm}}^{2}$), $I_{0}$ is the measured intensity of an empty sample chamber ($I_{0}$, ${W/\mathrm{cm}}^{2}$), *α* is the absorption coefficient (*α*, ${\mathrm{cm}}^{2}/\mathrm{mol}$), *C* is the CO concentration (*C*, ${\mathrm{mol}/\mathrm{cm}}^{3}$), and *L* is the absorption path length (*L*, cm) [10,11,12].

**Figure 4:**
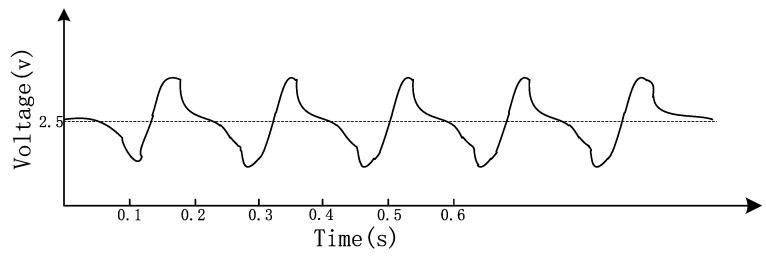
Sensor waveform by electrical modulation.

The level of CO concentration is represented at regular time intervals in the form of an electrical signal by a pyroelectric sensor. However, weak signal is vulnerable to interference by circuit. In order to improve the stability of the output signal, we adopt low frequency electric modulation techniques [13,14]. The data obtained by multiple experiments have proven that the modulation parameters T = 180 ms and D = 50% and they were adopted in the system to eliminate the fluctuations, as shown in Figure 4 [15].

**Figure 5:**
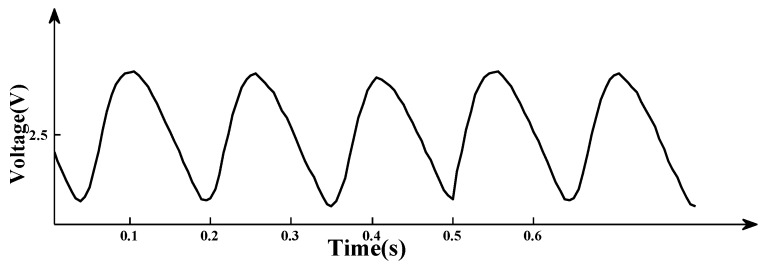
Waveform after digital filtered.

The output waveform is relatively stable after modulation.However, the modulated waveform inevitably contains noise components, so the digital filtering methods are used to filter the modulated waveform. In addition, compensation is needed for the impact of environment humidity on the gas concentration [16]. Having filtered and compensated the waveform on the basis of Figure 4, we obtained the final waveform shown in Figure 5.

According to the Lambert-Beer Law, attenuation of light intensity happens while the infrared goes through a gas cell containing CO. So, peak-to-peak of waveform value is associated with concentration of CO. Fixed CO concentration is measured by the system, according to correspondence between concentrations and peak-to-peak values, we can draw a curve fitting. Every waveform can get the corresponding concentration of CO referencing curve fitting.

## 3. System Network

The monitoring system is applied to industrial plants in the monitoring of CO leakage, which requires to take into account of toxicity and real-time monitoring. Wireless sensor networks (WSNs) have been extensively used for addressing such challenges [17].

There are many Wireless networks such as Zigbee, Wifi, Bluetooth and RFID. However, while Zigbee technology provides greater accuracy, its price is very high, and therefore it is discarded as an option, leaving active RFID and Wifi as the most viable alternatives. It also seems that Bluetooth is gaining ground once again when the Bluetooth presents incompatibilities with many devices. As for RFID, it can also provide one to one transmission, which does not form a data network [18].

Depending on the plant monitoring features and given the limitations in the range of radio transmission and network depth of common WSN solutions like Zigbee, it is infeasible to use them for such applications. Wifi promises to deliver high data rates over large areas to a large number of users, and forms network to transfer data directly to a computer and cell phone. So, Wifi is the most viable alternative.

**Figure 6:**
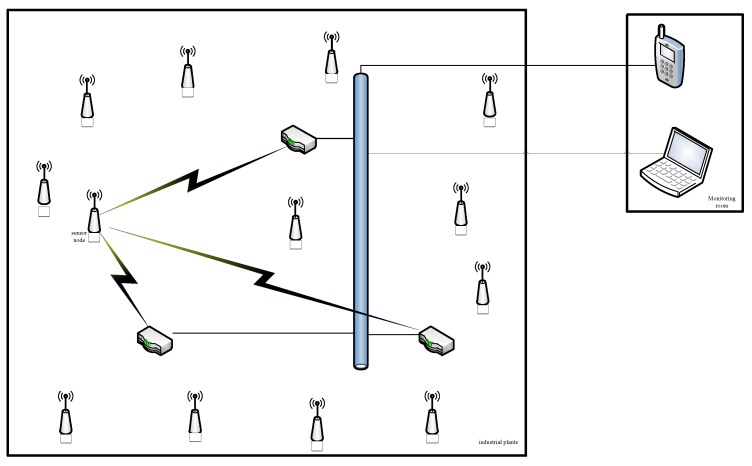
Architecture of the wireless sensor networks (WSNs).

The system network consists of station, Access Point (AP) and Personal Computer (PC), as shown in Figure 6. The station is a Wifi transceiver module, which connects the sensor node with RS232, communicating sensor node with AP. AP is also known as router, which collects data from many sensor nodes to PC or smartphone.

### 3.1. Network Configuration

#### 3.1.1. Base Station

The base station is based on Million Instructions Per Second program (MIPS) architecture, running at 360 MHz and featuring two wired Ethernet ports and one RS232 port. The device is equipped with programmable FLASH of 1 MB, random-access memory (RAM) of 8 MB, with maximum transmission distance up to 400 m when the environment is an open area. The TCP connections can be up to 32, which means the router can receive information from 32 base stations. The parameters of base station are as shown in Table 1. The base station connects the sensor node with RS232, and sends data to routers. In turn, routers can transmit instructions by base station to the sensor node to control the sensor switch. To reduce energy consumption, the radio parameters such as signal modulation and antenna were optimized [19].

**Table 1:** Parameters of base station.

System Message	Parameters	Wifi	Parameters
processor	MIPS program (32 bit, 360 MHz)	transmission distance	Max 400 m (open areas, 3 dB antenna)
RAM/Flash	8 M/1 M	frequency range	2.412∼2.484 GHz

#### 3.1.2. Access Point

The AP is also called the router, as shown in Figure 6, which is the intermediary base station and computers. The routers achieve each base station data transmission through the routers to the computer, and send instructions from the computer to the base station. The router is based on a 32-bit ARM7TDMI RISC processor, and running on linux operating system. The device is also equipped with programmable FLASH of 8 MB, random-access memory (RAM) of 32 MB, and peripheral function modules, which include Universal Asynchronous Receiver/Transmitter (UART), timers, programmable I/O ports and an interrupt controller.

### 3.2. Network Architecture

**Figure 7:**
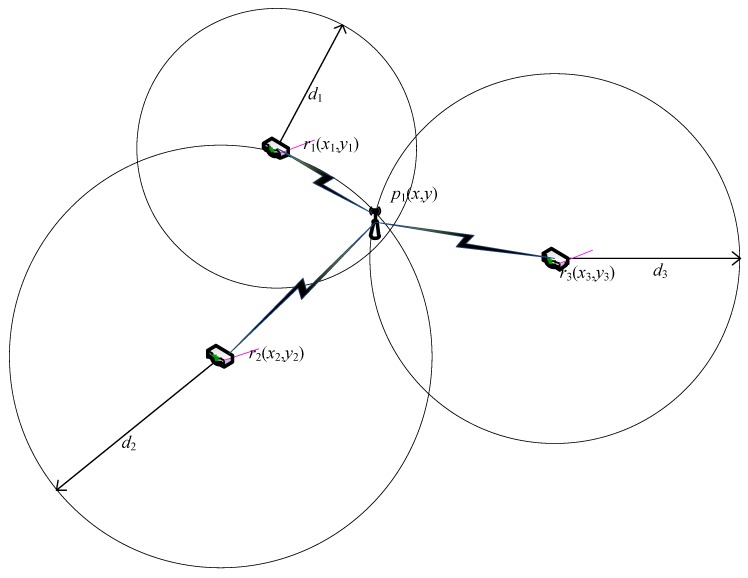
The schematic of algorithm.

The network architecture of the system is as shown in Figure 6. We place three routers in industrial plants. Each station has the Service Set Identifier (SSID) and mac address. The router receives the station mac address and signal strength, then acquires the distance to each base station based on the Receive Signal Strength Indicator (RSSI) [20]. Given the distance of every station to three routers, the triangle positioning method is proposed [21,22,23], and its principle is roughly shown in Figure 7.

We can build a plane Cartesian coordinate system for industrial plants, in which three routers are named *r*${}_{1}$, *r*${}_{2}$, *r*${}_{3}$ respectively, with coordinates (*x*${}_{1}$, *y*${}_{1}$), (*x*${}_{2}$, *y*${}_{2}$), (*x*${}_{3}$, *y*${}_{3}$). When routers receive the same mac address, they will define the sensor node as *p*${}_{1}$, and we will obtain the distance between *p*${}_{1}$ with every router as *d*${}_{1}$, *d*${}_{2}$, *d*${}_{3}$. If the coordinate of sensor node *p*${}_{1}$ is (*x*, *y*), the following formulas are formed:

$$\left\{\begin{array}{cc} \sqrt{{\left(x_{1}-x\right)}^{2}+{\left(y_{1}-y\right)}^{2}}=d_{1} & \;(2\text{a})\\ \sqrt{{\left(x_{2}-x\right)}^{2}+{\left(y_{2}-y\right)}^{2}}=d_{2} & \;(2\text{b})\\ \sqrt{{\left(x_{3}-x\right)}^{2}+{\left(y_{3}-y\right)}^{2}}=d_{3} & \;(2\text{c}) \end{array}\right.$$

From Equation (2a,b) , we can get (*x*, *y*) = (*x*${}_{12}$, *y*${}_{12}$), similarly, from Equation (2b,c), we can get (*x*, *y*) = (*x*${}_{23}$,*y*${}_{23}$), from Equation (2a,c), we can get (*x*, *y*) = (*x*${}_{13}$,*y*${}_{13}$); then,

(3) $$\left\{\begin{array}{c} \bar{x}=\frac{x_{12}+x_{23}+x_{13}}{3}\\ \bar{y}=\frac{y_{12}+y_{23}+y_{13}}{3} \end{array}\right.$$

So, the coordinate of sensor node *p*${}_{1}$ is ($\bar{x}$, $\bar{y}$). With the same method, we can get the sensor node. The address of the node is shown on the PC or smartphone in the form of coordinates. With the triangulation, we can locate the address of every sensor node; if one fails to work, the monitor react quickly. Of course, the new nodes added can be shown simultaneously.

### 3.3. Network Implementation

**Figure 8:**
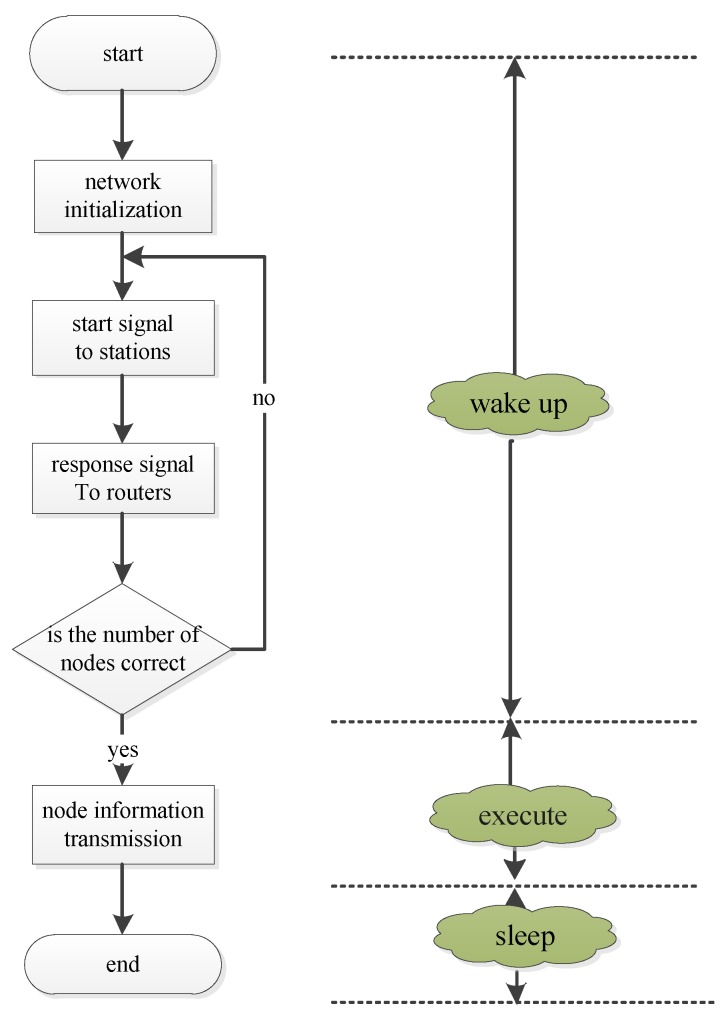
The network working status.

The sensor nodes obtain concentration of CO all the time. However, because of the power consumption of Wifi module [24], it is unwise with the Wifi module to open all the time. Additionally, it is not necessary to show the status of CO concentration continuously. Wifi module is designed with three different states: sleeping, wakeup, and execution. The state of sleeping lasts for 5 min, when 5 min is passed, one router issues a directive to every station for starting up. Every sensor node returns a response signal, and the position in coordinate system of PC or smartphone is located. So, we can determine the quality of the sensor and the newly added sensor nodes will be displayed in real time on a coordinate system. After a minute of data acquisition, the CO concentration of each node can be displayed. Then the network enters the state of Sleeping. Figure 8 shows the work state of the network.

## 4. Experimental Results

The purpose of this paper is to describe a real-time monitoring system for the monitoring of CO concentration. Based on this purpose, the system mainly consists of three parts: the sensor nodes, Wifi modules and routers. The sensor nodes monitor CO concentration a time point by Wifi modules; routers receive the information of each point, and transport it to a PC or smartphone.

To validate the accuracy of the system, we put the sensor nodes in a closed container, the temperature was kept between 28 ${}^{{}^{\circ}}$C and 29 ${}^{{}^{\circ}}$C and relative humidity was maintained at 50%–60%. First the sensor node was calibrated under the condition that the container was filled with nitrogen. Then the container was filled with CO under the conditions of different concentrations. The sensor node measured the level of these different concentrations. The measurement lasted three hours at each level of concentration and adjacent measurements were at an interval of one hour. The results are shown in Table 2.

The experimental data indicates that the minimum concentration detected by our sensor node is 15 ppm and the relative error is 4.2%, which means that the sensor node meets the requirements of the industry.

To verify the reliability of this monitoring system, we conducted the experiment in a plant (40 m long and 30 m wide) covered with pipes inside. The environmental state of industrial plants is as follows: all windows and doors were closed, the temperature was kept between 28 ${}^{{}^{\circ}}$C and 29 ${}^{{}^{\circ}}$C and relative humidity was maintained at 50%–60%. We placed a CO storage tank in industrial plants and the CO release rate is 5 ccm/min.

**Table 2:** Measurements of sensor node.

Concentration (ppm)	Measurements			Average	Relative Error
	(1)	(2)	(3)		
15	14.6	16.1	16.2	15.5	4.2%
30	30.5	30.9	31.4	30.9	3.1%
50	49.8	51.6	52.1	51.2	2.3%
100	97.7	101.4	106.5	102.5	1.87%
200	200.8	205.9	202.7	203.1	1.57%
300	299.2	306.6	303.3	303.0	1.01%

**Figure 9:**
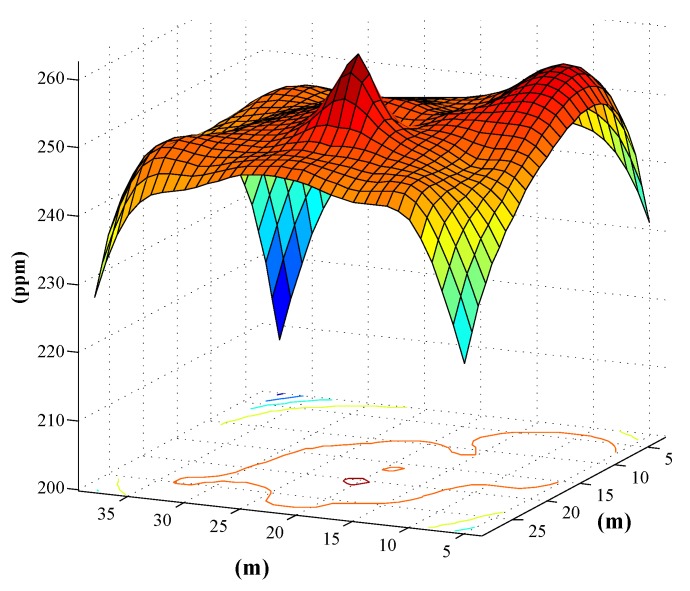
Predicted based on Gaussian Process (GP).

At a specific moment, every sensor node returned data to the PC, and we were able to acquire the concentration of CO in the place of sensor nodes. The spatial distribution of the indoor CO in the system was computed based on these sampling points, and the CO concentrations at points without sensors can be inferred from the distribution model. An effective Gaussian Process (GP) representation [25] is used for the data regression to establish the CO distribution model. Figure 9 shows the CO distribution model-based Gaussian Process, and the contour map can reflect the position of CO leakage points.

In order to verify the stability of the system, we ran 10 experiments on the same basis for one hour a day, and dozens of data about the maximum concentration of CO have been acquired. A simple normalization method is adopted, and these values are normalized according to the following equation:

(4) $$\begin{array}{cc} & y_{i}=\frac{x_{i}-\bar{x}}{s}\\ s.t. & \left\{\begin{array}{c} \bar{x}=\frac{1}{n}\sum_{i=1}^{n}x_{i}\\ s=\sqrt{\frac{1}{n-1}{\sum_{i=1}^{n}\left(x_{i}-\bar{x}\right)}^{2}} \end{array}\right. \end{array}$$

**Figure 10:**
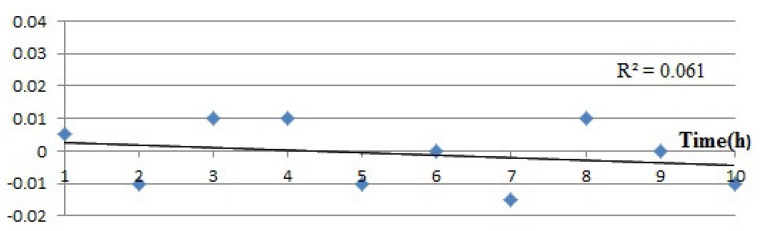
Stability test of the system.

Figure 10 shows the change of CO concentrations in the leakage point during 10 days’ continuous test. It can be inferred that the normalized values are within −0.02–0.01, in other words, the percentage of every value deviated from the mean is within −0.02–0.01, which indicates that the device has a better performance of stability.

## 5. Conclusions

We describe a real-time monitoring system for the concentration of CO based on Wifi WSN. For this purpose, a hardware system is designed to detect the CO concentration, and a low-frequency modulation method is proposed to improve the stability and accuracy. A wireless network using Wifi technology can transmit the information of sensor nodes, and the triangulation achieves the function of monitoring the location and operational status of each node. The system is deployed within an experimental environment, and the experimental results show that the system meets the stability and accuracy expectations.
